# Study on the critical stable height of vertical excavation in rocky foundation pit within layered structural plane

**DOI:** 10.1038/s41598-024-63063-2

**Published:** 2024-05-28

**Authors:** Ziguang Zhang, Xueping You, Cheng Zhang, Wanyu Li, Mengqing Zhang

**Affiliations:** Anhui Province Key Laboratory of Building Structure and Underground Engineering, Anhui Jianzhu University, Hefei, China

**Keywords:** Engineering, Civil engineering

## Abstract

The structural plane characteristic was the most critical factor for determining the self-stability ability of deep foundation pit vertical-rock-wall in layered rock stratum. Multiple methods such as model testing, numerical calculation, and theoretical calculation were utilized comprehensively in this paper. The self-stabilizing control effect on the deep foundation pits vertical-rock-wall that under the different structural plane inclination angle (*α)* and under the different structural plane strength was systematically studied. The results indicated that the overall variation trend of "Sharp decrease ~ Slow decrease ~ Slow increase ~ Sharp increase" in the symmetrical distribution for the self-stability critical height (*Hcr)* varied with the gradually increasing of *α* was presented. Meanwhile, the variation trend of "continuously decreasing and rapidly decreasing first, and then slowly decreasing and tending to stabilize" with the structural plane strength reduction coefficient *(k*). The key factor to control the self-stability of the deep foundation pit vertical-rock-walls lied in fully grasping and utilizing the basic characteristics of rock structural planes. The research results of this paper provided the theoretical basis for scientifically determining the safety level and designing reasonable support structures of the deep foundation pit vertical-rock-walls in layered rock stratum.

## Introduction

The self-stable height of the deep foundation pit vertical-rock-wall is the important basis for evaluating the safety level and designing the support structures. The relevant theories of the self-stable analysis mainly start from the deep foundation pit in soil strata and have achieved rich research results^1–4^. However, there is relatively a few research on the self-stability characteristics of deep foundation pits in rock strata^5^. The majority of scholars still study the self-stability characteristics of the deep foundation pits in rock strata based on the relevant theories of that in soil strata. Due to the significant differences between the rock mass and the soil mass^6–9^, it is obviously biased to use the foundation pit related theories in soil strata to study that in rock strata^10,11^. The correct understanding and reasonable evaluation of the self-stability characteristics of the foundation pit vertical-rock-wall in rock strata have important practical significance and theoretical value.

The analysis of instability modes is the basis for evaluating the self-stability characteristics of the deep foundation pit vertical-rock-wall. The characteristics of the rock structural planes are the most critical factors determining the self-stability characteristics of the deep foundation pit vertical-rock-wall in layered rock stratum. In the 1860s, Culmann^12^ derived the formula for calculating the critical instability height of vertical sidewalls based on the force equilibrium conditions. Subsequently, Terzaghi^13,14^ modified the Culmann method and developed a series of theoretical formulas for the critical instability height of vertical sidewalls. Pufahl^15^ derived the self-stable critical height of the vertical excavation foundation pit by calculating and analyzing the conditions under which the sliding wedge was in the limit equilibrium state. Based on the principle of capacity balance, Chen etc.^16,17^ obtained upper bound solutions for the self-stability critical height of slopes in the homogeneous stratum and the heterogeneous stratum respectively. Moussaei etc.^18^ and Li etc.^19^ studied the influence of factors such as dip angle and spacing of layered joints on the failure of surrounding rock through model experiments and numerical simulations. Structural property is one of the fundamental properties of the rock mass. Rock mass is composed of the various types of structural planes and the complex geometry and variable scale structural bodies that formed by cutting structural planes. Rock mass usually exhibit the characteristics of non-homogeneity, discontinuity and anisotropy. According to the different mechanical properties, wall morphology, strength, and internal filling conditions, rock mass structural planes are generally divided into hard structural planes and weak structural planes. The former mechanical property depends on the wall roughness and strength, while the latter is related to the filling material, mineral arrangement etc.^20^. The presence of weak structural planes considerably diminishes the mechanical strength of the rock mass. The weak structural plane often determines the mechanical properties and deformation characteristics of rock mass, and which is the controlling factor for the stability of rock mass engineering^21–23^. Due to the presence of structural planes that are contributed by various factors, the rock mass failure mechanism and stabilization issues are very complex in practical engineering. Scholars have studied the influence of structural plane parameter characteristics on the self- stability characteristics of rock foundation pit slopes from different perspectives^24–27^. Hammah etc.^28^ applied the finite element nodal network to study the failure mechanism of rock slopes and the effect of different scales on slope stability. Shamekhi etc.^29^ established a slope rock mass fracture network model which considered the variability of geometric parameters, quantified the contribution of each geometric parameter, and then predicted the probability of slope failure. Zhuang etc.^30^ proposed a rock slope reliability evaluation method based on the discrete fracture network (DFN) model and Latin hypercubic sampling method, which integrated the uncertainty of the strength parameters of the rock joints, the variability of the geometric parameters, and the randomness of the location. Lin etc.^31^ proposed a numerical model that considers the friction coefficient, bonding stiffness, and strength of the constituent materials, as well as the stiffness and strength of interlayer bonding. Guo etc.^32^, Dong etc.^33^, Wang etc.^34^ and Li etc.^35^ used different methods to reveal the failure mechanism of rock slopes in weak and hard interlayered.

**Figure 1:**
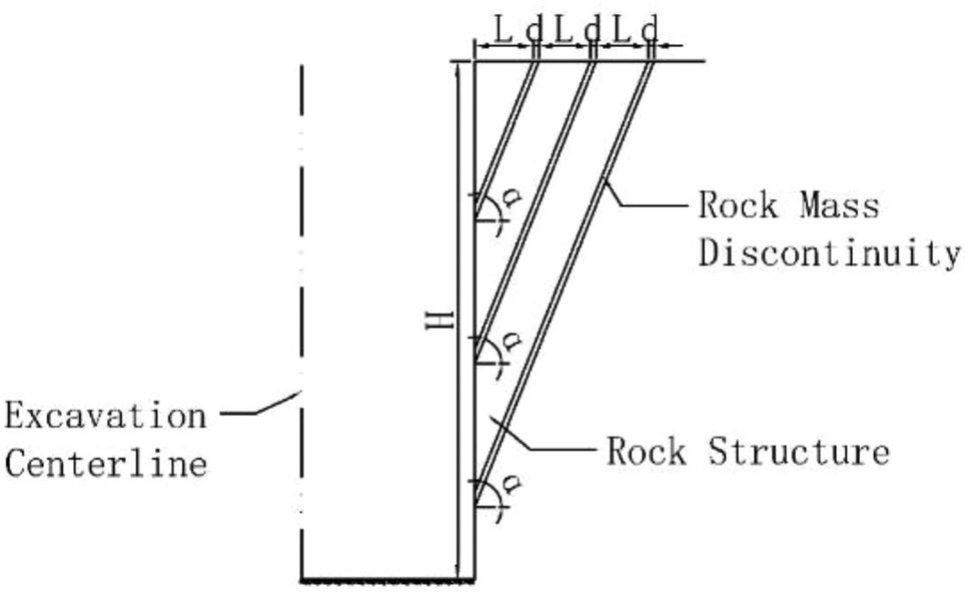
The analysis model of the FPVRW in layered rock stratum.

In summary, the current research on the influence of structural plane characteristics on the stability of deep foundation pits vertical-rock-wall (FPVRW) in layered rock stratum is mainly carried out in conjunction with specific engineering, and only a few working conditions are analyzed. The calculation result data is not continuous enough, and the randomness of the calculation conclusion is high. In view of this, based on the basic prototype of the deep foundation pit of Ningxia Road metro station in Qingdao in this paper^36^, and the following assumptions and simplifications are made: (1) The excavation model of the foundation pit is simplified into a plane model; (2) The direction of the rock mass structural plane is consistent with the direction of the foundation pit sidewall; (3) The inclination of the structural plane towards the excavation free face of the foundation pit and tilts inward towards the free face; (4) The structural planes are interconnected and parallel with equal spacing. The analysis model of the self-stabilizing control effect of the structural plane on the FPVRW in layered rock stratum was shown in Fig. 1. In this figure, *H* represented the height of the FPVRW, which was also the excavation depth of the foundation pit; *D* represented the excavation width of the foundation pit; *α* represented the internal inclination angle of the rock structural plane, and *d* represented the thickness of the rock structural plane;* L* represented the thickness of the rock mass structural body. The testing similar materials are prepared according to a geometric dimension ratio of 1:50, and the self-stabilizing control effect model test of the structural plane characteristics on the FPVRW is adopted firstly. And then, numerical test is used to perform proportional calculation and analysis on the self-stability characteristics of the FPVRW. Next, the reasonable and simplified theoretical calculation model for the self-stabilizing control effect of the FPVRW is constructed, and corresponding theoretical calculation equations are derived. Finally, the self-stabilizing control effect of the FPVRW under the different structural plane inclination angle and the different structural plane strength is systematically studied, and the reasonable suggestions are proposed.

## Materials and methods

### Model test

#### Similar material preparation

The quartz sand and the barite powder were selected as the aggregate substances of the similar materials, and the mixture of gypsum and cement were selected as the cementitious material in model tests. The quartz sand was made of the medium sand with the fineness specification of 20–35 mesh and the density of approximately 2.73 g/cm^3^. The barite powder made of the powder with the fineness specification of 400 mesh and the density of 4.2 g/cm^3^.The model gypsum was used for gypsum, and C42.5 benchmark cement was used for cement. According to the similarity principle, the geometric similarity ratio (*C*_*l*_) of the deep foundation pit model test was taken as 50, and the severity similarity ratio (*C*_*γ*_) of the rock mass material was taken as 1.2. The elastic modulus similarity ratio (*C*_*E*_) and the cohesion similarity ratio (*C*_*c*_) were both taken as 60, and Poisson's ratio similarity ratio (*C*_*μ*_) and the internal friction angle similarity ratio (*C*_*φ*_) were both taken as 1.0. According to the actual foundation pit project of Ningxia Road metro station in Qingdao, the parameter values related to similar materials in model test were shown in Table 1.

**Table 1:** The material parameters of model test.

Parameter type		*E*/(MPa)	*μ*	*c*/(MPa)	*φ*/(°)	*γ*/(KN·m^−3^)
Similarity ratio		*C*_*E*_ = 60	*C*_*μ*_ = 1.0	*C*_*c*_ = 60	*C*_*φ*_ = 1.0	*C*_*γ*_ = 1.2
Rock mass structure	Raw material	5000	0.25	600	35	22.5
	Similar materials	83.33	0.25	10	35	22.5
Rock structural plane	Raw material	50	0.38	100	30	20.5
	Similar materials	0.83	0.38	1.67	30	1.96

Similar materials in the model tests were determined through orthogonal experiments using mechanical performance testing for two indicators, which were the mass ratio of the quartz sand to the barite powder and the mass ratio of the aggregate to the cementitious material. There were the total of 16 test conditions. Four test conditions of 8:1, 9:1, 10:1, and 10:0 were taken for the mass ratio of the quartz sand to the barite powder, and four test conditions of 20:1, 25:1, 30:1, and 40:1 were taken for the aggregate to the cementitious material. Six samples of *Ф*50 mm × 100 mm and four samples of *Ф*61.8 mm × 20 mm were made per group used for uniaxial compression tests and direct shear tests respectively. The test results of physical and mechanical parameters of the similar materials in the model tests were shown in Table 2. For the selection of similar materials, it was impossible to simultaneously satisfy the similarity of all experimental parameters. For one parameter, selecting material A was better, while for another parameter, material B might be more suitable. The rock mass structure of this model test was composed of similar materials with the mass ratio of 10:1 for crystalline sand and the bone glue mass ratio of 30:1. While the rock structural planes were prepared using the crystal sand mass ratio of 10:0 and the bone glue mass ratio of 30:1.

**Table 2:** The test results of physical and mechanical parameters of similar materials.

S/N	Material ratio		Testing parameters				
	Q.S:B.P	A:C.M	*E*/(MPa)	*μ*	*c*/(MPa)	*φ*/(°)	*ρ*/(g·cm^−3^)
1	8:1	20:1	34.79	0.27	52.448	35°	1.911
2	8:1	25:1	27.53	0.28	43.4335	34°	1.849
3	8:1	30:1	23.92	0.3	36.8775	34°	1.876
4	8:1	40:1	6.74	0.31	37.697	30°	1.866
5	9:1	20:1	24.78	0.27	51.6285	36°	1.858
6	9:1	25:1	19.49	0.33	40.1555	34°	1.877
7	9:1	30:1	26.72	0.35	30.3215	34°	1.845
8	9:1	40:1	16.67	0.38	41.7945	25°	1.882
9	10:1	20:1	44.3	0.17	40.975	37°	1.848
10	10:1	25:1	34.16	0.28	32.78	34°	1.854
11	10:1	30:1	51.36	0.3	9.0145	35°	1.845
12	10:1	40:1	13.3	0.33	7.3755	23°	1.827
13	10:0	20:1	37.64	0.23	11.473	32°	1.749
14	10:0	25:1	27.43	0.29	5.7365	31°	1.711
15	10:0	30:1	19.36	0.32	1.639	29°	1.701
16	10:0	40:1	5.58	0.40	0.8195	25°	1.641

*Q.S:B.P* The mass ratio of the quartz sand to the barite powder; *A:C.M* The mass ratio of the aggregate to the cementitious material.

#### Test instruments and equipment

**Figure 2:**
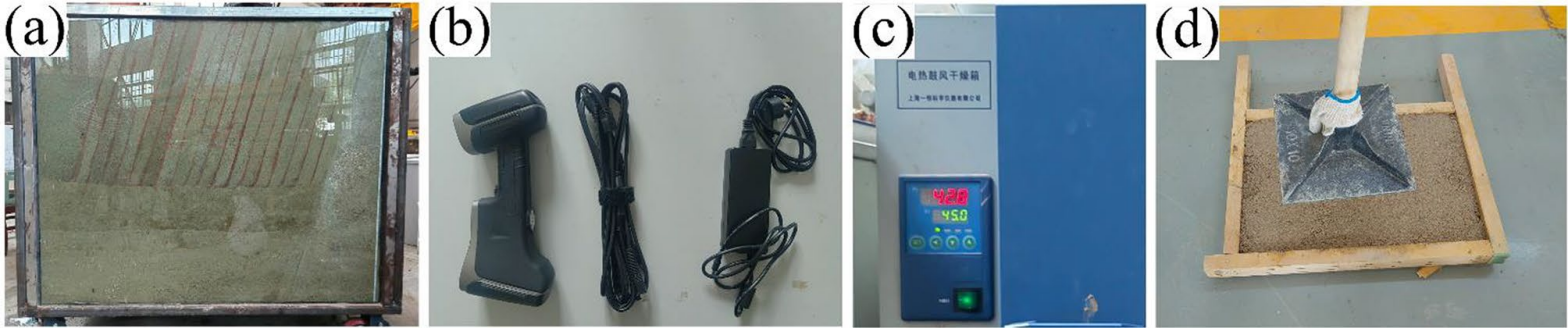
Test instruments and equipment: (**a**) The self-made movable model box; (**b**) 3D laser scanner and its supporting equipment; (**c**) Constant temperature blast drying oven; (**d**) Earth rammer.

The model test equipment mainly consisted of four parts which were the self-made movable model box, the 3D laser scanner and its supporting equipment, the constant temperature blast drying oven and the earth rammer, as shown in Fig. 2. The length × width × height of the model box was 1.5 m × 1 m × 1.2 m. The four walls of the model box were composed of three steel plates and one tempered glass plate, and the steel plate corresponding to the glass plate was designed to the integral opening and closing door for the convenience of stacking and excavation of test blocks. The bottom plate of the model box was composed of steel plates, and the movable pulley block were installed below the bottom plate. All steel plates in the model box were made of integral steel plates, and the row of 1 mm diameter holes were set every 30 cm on the steel plates. The steel plates and the tempered glass plate were welded through corner columns. The scanner adopted FreeScan UE Pro composite 3D scanner with the accuracy of 0.02 mm. The scanner supporting equipment included a power adapter connection cable, marking points, and the USB cable with 4 interfaces for connecting the computer, the power adapter, etc. The constant temperature blast drying oven with the size of 0.83 m × 0.59 m × 0.74 m, the temperature range of 10–300 °C, the temperature resolution of 0.1 °C, and the normal heating rate of 3–5 °C/min. The earth rammer consisted of a rammer head and a long handle, with the total weight of 10 kg.The tamping head was integrally formed by cast steel, and the tamping surface size was 25 cm × 25 cm.

#### Test process

The implementation process of the model testing method was completed in five steps.

**Figure 3:**
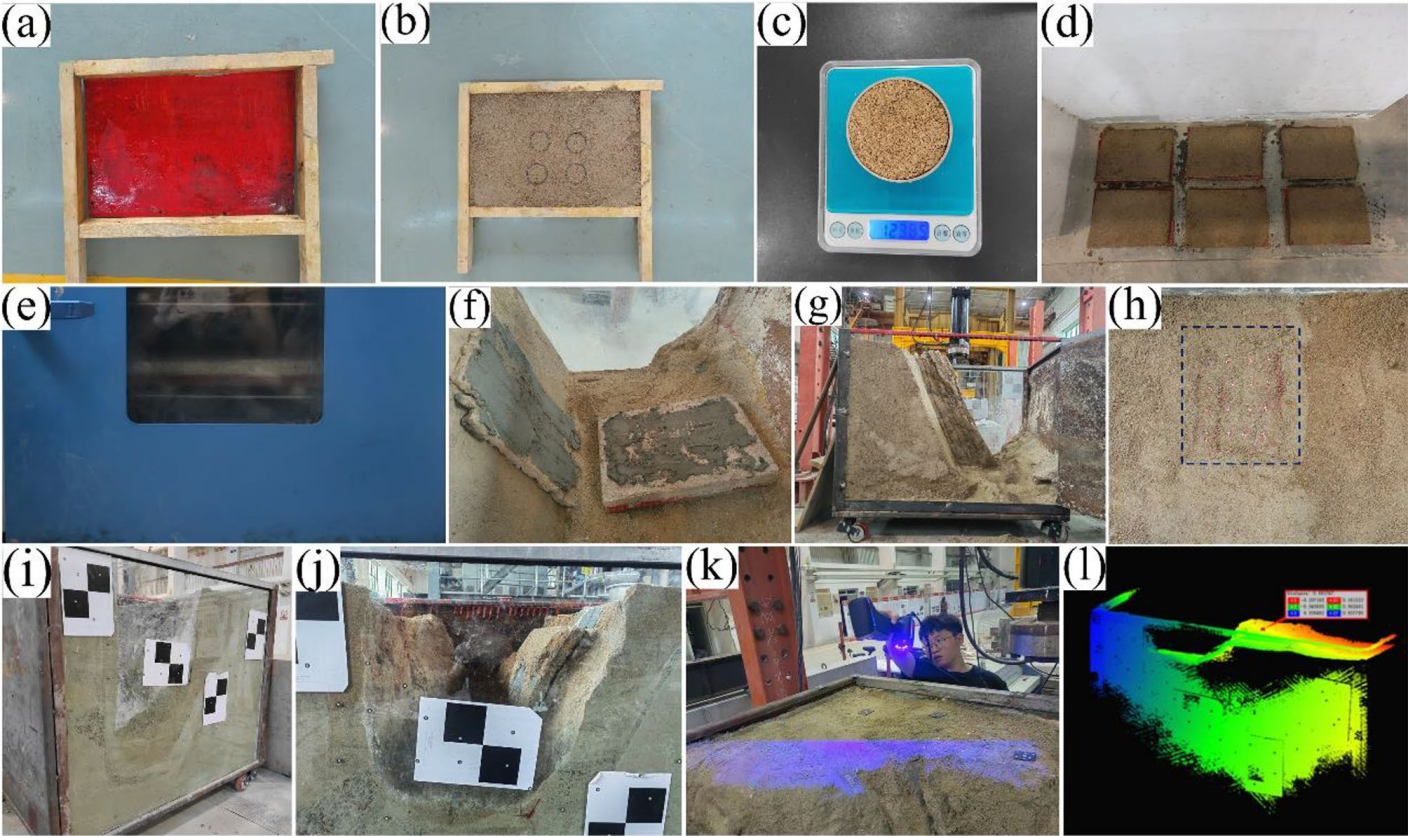
Test process: (**a**) Compaction test mold; (**b**) Material compaction; (**c**) Ring knife sampling; (**d**) Density test; (**e**) Making test blocks; (**f**) Oven drying; (**g**) Stacking test block; (**h**) Paste test blocks; (**i**) Layout of monitoring points; (**j**) Coordinate transformation points; (**k**) Layered excavation; (**l**) scanning.

Step 1: Compaction test. Before making the simulated test blocks of rock mass structure, the compaction test was first conducted to determine the compaction standard during the production of the test blocks. The compaction test mold was composed of four detachable and assembled rectangular wooden strips with the length × width × height of 50 cm × 8 cm × 8 cm, as shown in Fig. 3a. Firstly, the stirred similar material was filled into the mold according to a certain thickness. Then, the filled material was compacted by the earth hammer with the falling freely from the height of 10 cm above the material surface, as shown in Fig. 3b. After the material was compacted every time, the middle part of the material was sampled by the ring cutter, and the material was weighed by an electronic scale with the accuracy of 0.001 g, as shown in Fig. 3c,d. The sample density value was calculated, and the curve of the sample density continuously varying with the compaction times increase was drawn. When the density remained basically unchanged, the number of the compaction times was used as the standard number for the production of the model test blocks.

Step 2: Making test blocks. The similar material test blocks of the rock mass structure were made according to the structural body blocks and structural plane blocks separately. The production of the test block adopted the same method as the compaction test, and was completed by the same person, the same hammer, the same height, and the same number of the compaction times, as shown in Fig. 3e. After the production of the test block had been completed, dried it in a natural environment for 12 h at first, and then dried it in the constant temperature blast drying oven, as shown in Fig. 3f. During the drying process, the sample was weighed at a certain time interval to obtain its density, until the density of the test block reached the density required for similar material preparation, and then it was taken out of the oven.

Step 3: Stacking test blocks. The dried test block was placed in the model box according to the designed inclination angle, as shown in Fig. 3g. The rock mass structural body test block and the structural plane block were bonded with cement slurry, as shown in Fig. 3h.

Step 4: Layout of the monitoring points. Three rows surface subsidence monitoring points with the spacing of 50 mm × 50 mm were deployed in the direction parallel to the side-wall of the proposed excavation foundation pit, as shown in Fig. 3i. The scattered laser scanning markers were placed among the monitoring points for data collection, and the targets were pasted onto the glass on the side of the model box for coordinate transformation during data collection, as shown in Fig. 3j.

Step 5: Excavation and monitoring. Model test was excavated in layers with a thickness of 4 cm per layer until the instability occurred, as shown in Fig. 3k. After each layer of excavation was completed, the monitoring points were scanned in time, as shown in Fig. 3l. Each measurement results of surface subsidence monitoring points were converted into the same coordinate system using the coordinates convert points, and the surface subsidence value of each monitoring point after each layered excavation completion was obtained using point ranging measurement methods.

### Numerical test

**Figure 4:**
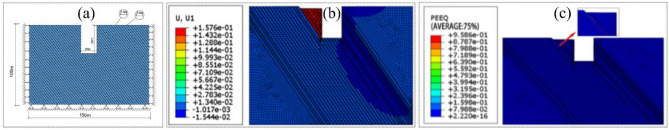
The numerical calculation results: (**a**) Number test model; (**b**) Displacement map; (**c**) Plastic zone map.

The numerical experiment was completed using ABAQUS numerical calculation software. The two-dimensional plane analysis model was constructed based on ideal elastic–plastic solid elements for both the rock mass structure and structural planes. The rock mass structure bodies and the structural planes were connected by contact units. The upper boundary of the numerical experiment model was taken to the earth's surface. The lower boundary was taken to 3 times of the excavation depth below the bottom of the foundation pit, and the horizontal distance between the model boundary and the side wall of the foundation pit was greater than 3 times the excavation depth. The upper boundary of the experimental model was free. The horizontal constraints were applied to the left and the right boundaries of the model, while the vertical constraints and the horizontal constraints were applied to the bottom boundary. The grid size of the foundation pit was set as 2.0 m. The improved Mohr–Coulomb constitutive model was adopted for rock mass structure. The initial stress only considered the influence of the self-weight of the rock mass structure, without considering the other factors such as groundwater and earthquakes. Taking the deep foundation pit in layered rock stratum as an example for detailed explanation, which the inclination angle *α* was 60° and the thickness *d* was 0.5 m, the thickness *L* was 2.5 m, the excavation width *D* was 20 m and the proposed excavation depth *H* was 36 m, as shown in Fig. 7a. The length and the width of the model were taken as 150 m × 100 m, respectively. When dividing the numerical calculation grid, the global seed setting was used with the size of 2 m. The total number of grid units was 5939, including 5832 quadrilateral units and 107 triangular units. The total number of grid nodes was 6033. The crustal stress balance equation was solved by the complete Newton solver, which iterated multiple times for each incremental step under each analysis step to achieve convergence, and then obtained the convergence solution under that analysis step. The initial incremental step of the crustal stress balance analysis was defined as 0.1, and the minimum incremental step was 1e − 7. The maximum incremental step was defined as 1, and the maximum number of incremental steps was 2000. In the load module, the crustal stress analysis step was used and the physical module was selected. The weight of the rock mass structural body and the structural plane were set in the vertical direction. The interaction between the rock structural plane and the structural body adopted the universal contact with friction. The normal mechanical behavior adopted the hard contact, while the tangential mechanical behavior adopted the penalty function with the friction coefficient of 0.8. The foundation pit was excavated in layers, with the total of 12 layers excavated at the depth of 3 m per layer. The distribution map of the stress, the deformation, and the plastic zone could be obtained during the entire numerical calculation process from the beginning to the non-convergence. Meanwhile, the magnitude of values such as stress and deformation could be extracted, as shown in Fig. 4.

### Theoretical research

**Figure 5:**
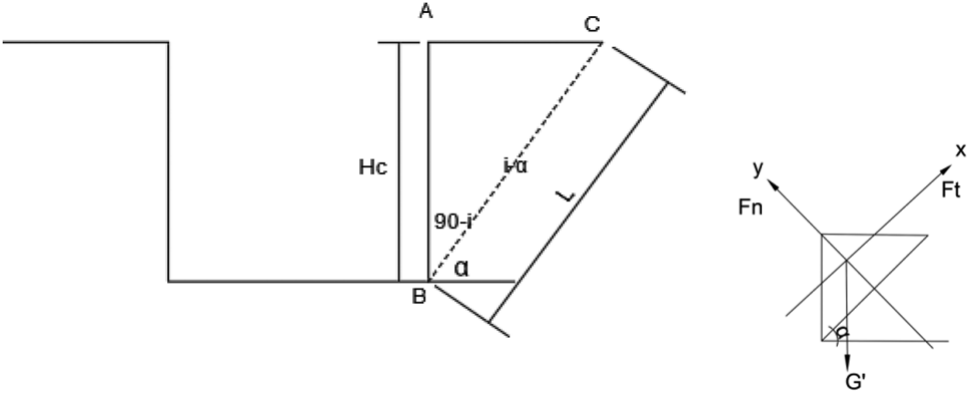
The theoretical analysis model of self-stable characteristics for the FPVRW.

The theoretical research on the self-stability characteristics of the FPVRW in layered rock stratum was simplified as follows^36^: (1) The fracture surface of the FPVRW was a single plane sliding rock structural plane, and the fractured body was a rigid body. (2) The rock structural plane is connected and regardless of the thickness. (3) Horizontal distribution of the ground surface. Based on the above simplification, the theoretical analysis model for the self-stability characteristics of the FPVRW in layered rock stratum could be constructed as shown in Fig. 5. In this figure, AB represented the height of the FPVRW, and its value was set as *Hr*. BC represented the rock structural plane of the deep foundation pit, and its length was set as *s*. ABC represented the potential fracture body of the FPVRW, and its unit weight set as*γ*, while its gravity set as *G*.

Assuming that the cohesion of the deep foundation pit structural plane was *c*, and the internal friction angle was *φ*. The angle between the structural plane and the horizontal plane of the formation was *α*, that is the inclination angle of the structural plane was *a.* The normal stress component of ABC on BC was *F*_*N*_, while the tangential stress component was *F*_*T*_.

According to geometric conditions, the relationship between the length* s* of the structural plane BC and the height *H* of the deep foundation pit vertical-rock-wall AB satisfied the Eq. (1). The relationship between length s and height H satisfied Eq. (1).

1 $$\text{s}=\frac{H}{sin\alpha }$$

The expression for the gravity *G* of the potential fracture body in deep foundation pits was shown in Eq. (2).

2 $${\text{G}}{\prime}=\frac{1}{2}\upgamma {\text{H}}^{2}\text{cot}\alpha $$

From the force equilibrium relationship of ABC in the x direction and y direction of BC, it could be obtained that normal stress component *F*_*N*_ and tangential stress component *F*_*T*_ were expressions (3) and (4).

3 $$\sum {F}_{y}=0: {F}_{N}={G}{\prime}cos\alpha $$

4 $$\sum {F}_{x}=0: {F}_{T}={G}{\prime}sin\alpha $$

When the FPVRW was in the critical self-stable state, the tangential stress component *F*_*T*_ was equal to the shear strength of the sliding body, and thus Eq. (5) could be obtained.

5 $${F}_{T}= {F}_{N}\text{tan}\varphi +\text{ c}.\text{s}$$

When Eqs. (1) to (4) were taken into Eqs. (5), Eq. (6) could be obtained.

6 $$\frac{1}{2}\upgamma {H}^{2}\text{cot}\alpha \text{sin}\alpha = \frac{1}{2}\upgamma {H}^{2}\text{cot}\alpha \text{cos}\alpha \text{tan}\varphi +\text{ c}. \frac{H}{sin\alpha }$$

When organizing Eq. (6), Eq. (7) could be obtained.

7 $$H=\frac{2c}{\gamma \text{cos}\alpha (\text{sin}\alpha -cos\alpha \text{tan}\varphi )}= \frac{2c\times cos\varphi }{\gamma \times \text{cos}\alpha \text{sin}(\alpha -\varphi )}$$

Equation (7) was the calculation formula for *Hcr*, which was also Eq. (8).

8 $${H}_{cr}=\frac{2c\times cos\varphi }{\upgamma \times \text{cos}\alpha \text{sin}(\alpha -\varphi )}$$

## Results

**Figure 6:**
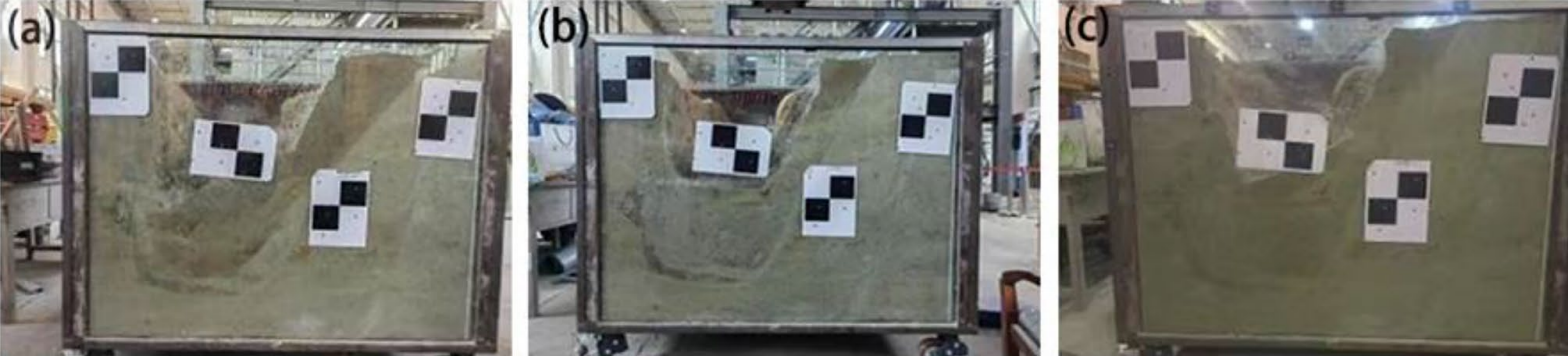
The model test results: (**a**) α = 50°; (**b**) α = 60°; (**c**) α = 70°.

**Figure 7:**
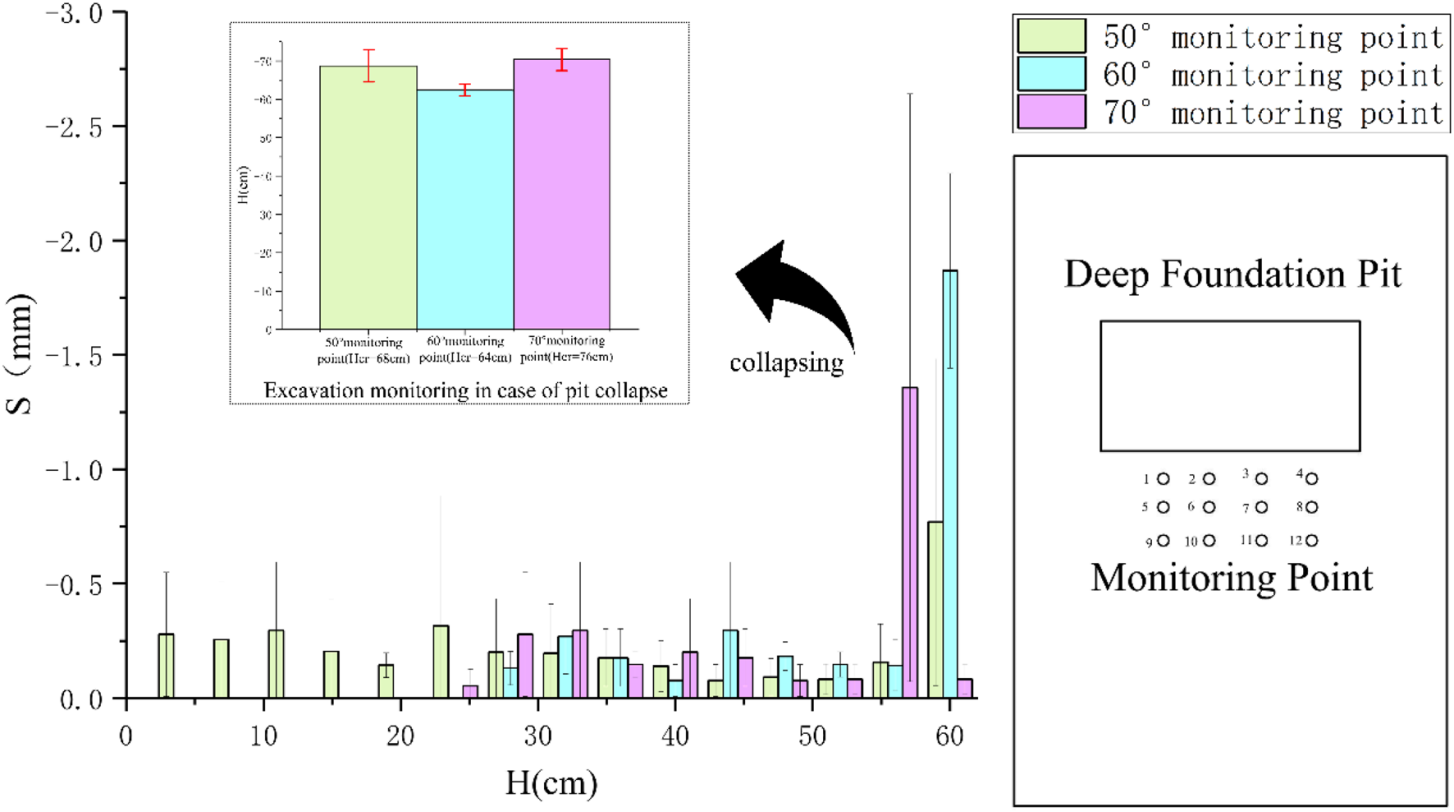
The curve of the surface settlements at the monitoring point of the deep foundation pit model test changed with excavation depth.

Rock structural plane inclination angle (*α*) took three working conditions of 50º, 60º, and 70º in sequence, and the cohesion (*c*) and internal friction angle (*φ*) of the rock structural plane under various working conditions were all taken as 100 kPa and 30° in model test. Experimental results indicated that the depth at which the vertical-rock-wall of the foundation pit model lost stability, i.e. the critical height (*Hcr*) of the FPVRW, was 64 cm, 60 cm and 72 cm respectively, when the inclination angle (*α*) of the structural plane was 50º, 60º, and 70º in sequence, as shown in Fig. 6. The layout of monitoring points was shown on the right side of Fig. 7. On the left side of Fig. 7, the bar chart below showed the displacement changes and errors of monitoring points with excavation depth before collapse of three different inclined structural planes in foundation pits, while the small bar chart above showed the mean displacement mutation and its error graph when the foundation pit suddenly collapses.

**Figure 8:**
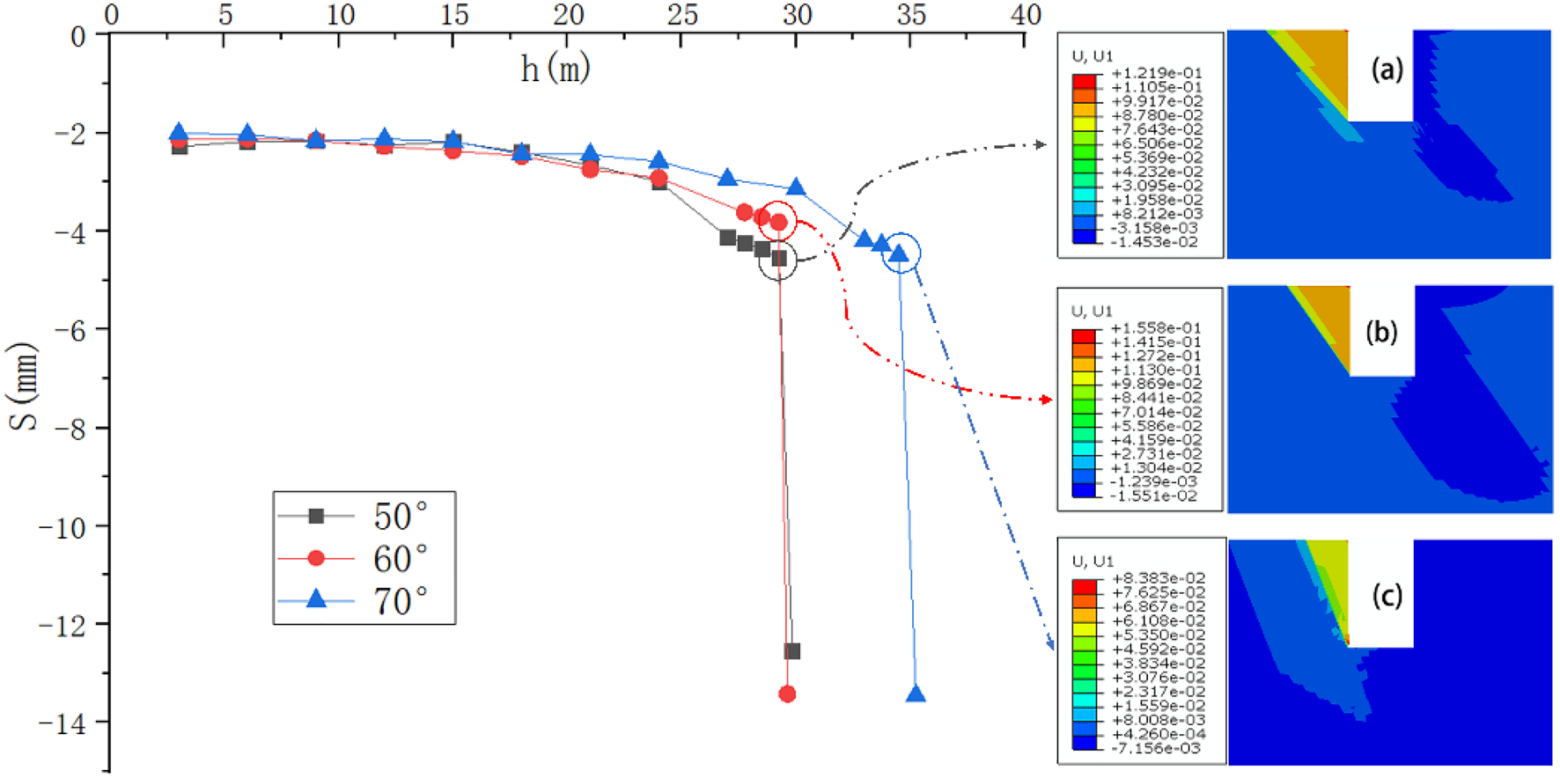
The *S–h* curve of characteristic point in deep foundation pit changing.

**Figure 9:**
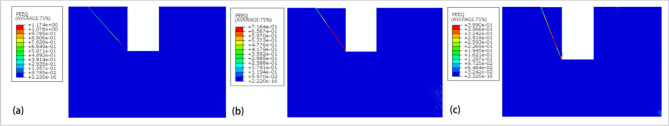
The numerical calculation of plastic zone connectivity diagram: (**a**) α = 50º; (**b**) α = 60º; (**c**) α = 70º.

The numerical calculation selected the intersection of the inclined vertical-rock-wall free surface and the surface in the structural plane of the foundation pit as the characteristic point for the analysis of the self-stability control effect of the vertical-rock-wall of the deep foundation pit. The vertical displacement (S) of the characteristic point of the deep foundation pit changes with the excavation depth h, and the excavation depth corresponding to the sudden change point of the *S–h* curve was used as the critical height (*Hcr*) value of the self-stability of the vertical rock wall of the deep foundation pit. Drew the curve of the vertical displacement *S* of the deep foundation pit characteristic points as the function of the excavation depth (*h*), and the excavation depth corresponding to the sudden change point of the *S–h* curve was used as the critical height (*Hcr*) for the self-stability of the FPVRW. For the deep foundation pits with the rock structural plane cohesion *c* and the internal friction angle (*φ*) of 100 kPa and 30°, and the inclination angles *α* of 50°, 60°, and 70° respectively, the *S–h* curve of the feature points was shown in Fig. 8. The critical height (*Hcr*) of the FPVRW was 29.84 m, 29.63 m and 35.59 m, respectively. At the moment of the *S–h* curve catastrophe, the displacement cloud map and the plastic zone cloud map of the foundation pit were shown in Figs. 8 and 9, respectively.

Three research methods were used, namely the model testing method, the numerical calculation method, and the theoretical calculation method. When the cohesion *c* and the internal friction angle (*φ*) of the rock structural planes was 100 kPa and 30°, and the inclination angles *α* was 50°, 60°, and 70° in sequence, the calculation results of the self-stability critical height (*Hcr*) was shown in Table 3. The research results obtained by the three research methods were basically consistent.

**Table 3:** The calculation results of the *Hcr*.

Inclination angle*α*/(°)	50	60	70
Model experimental method	32.00	30.00	36.00
Numerical calculation method	29.84	29.63	35.59
Theoretical calculation method	35.02	30.79	35.02

### The control effect of the rock structural plane inclination angle

**Figure 10:**
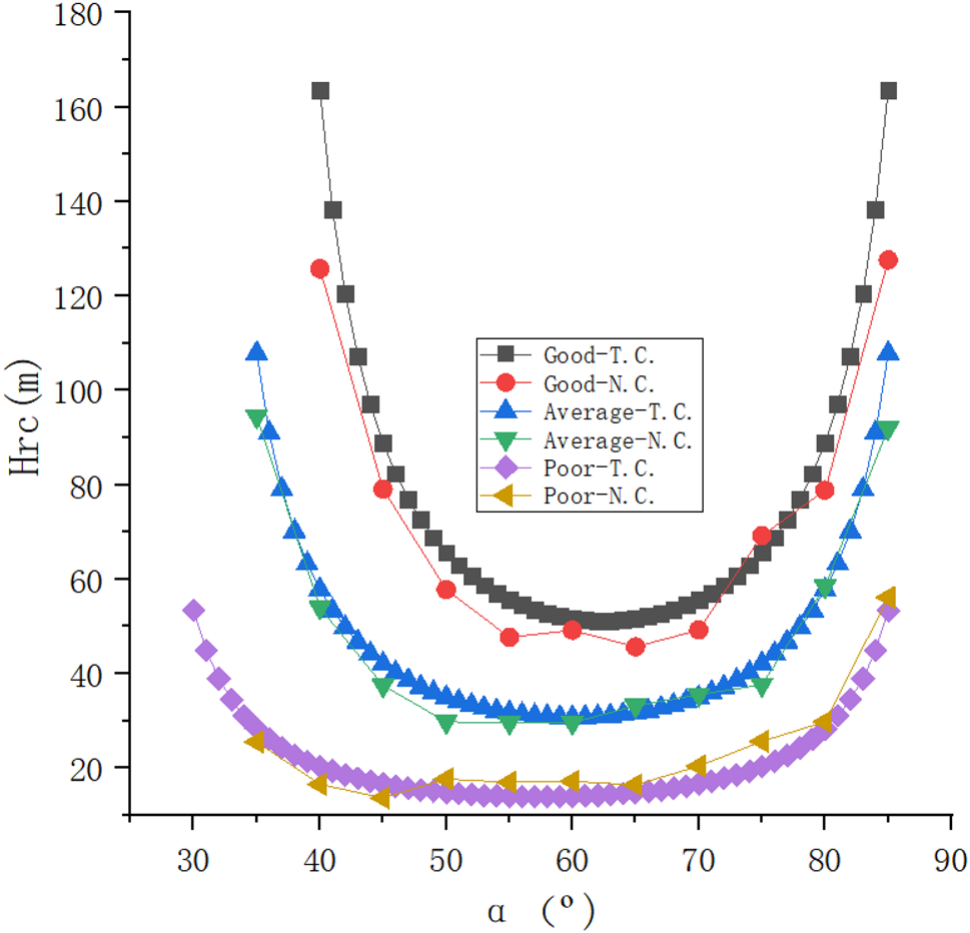
The change distribution characteristics of the *Hcr* varied with *α*.

The rock structural planes occurrence was taken as inward inclined structural planes with angles (*α*) of 30º, 35º, 40º, 45º, 50º, 55º, 60º, 65º, 70º, 75º, and 80°in sequence. Three structural plane working conditions were adopted, namely the cohesion force (*c*) and internal friction angle (*φ*) were taken as 150 kPa and 35°, 100 kPa and 30°, 50 kPa and 25°in sequence. For the narration convenience, they were respectively referred to as good combination, average combination, and poor combination, as shown in Table 4. The distribution characteristics of the *Hcr* varied with the gradually increasing of*α*were shown in Fig. 10.

**Table 4:** The combination degree of rock structural plane.

Combination degree	Cohesion force *c/*(*kPa*)	Internal friction angle *φ/*(°)
Good combination	150	35
Average combination	100	30
Poor combination	50	25

The conclusions could be drawn from Fig. 10 that the overall variation pattern of "Sharp decrease ~ Slow decrease ~ Slow increase ~ Sharp increase" in a symmetrical distribution for the *Hcr* varied with the gradually increasing of structural plane inclination angle(*α*) was presented. Under the same conditions of structural plane inclination angle(*α*), the worse the shear strength, the smaller the *Hrc*. For the deep foundation pit in layered rock stratum with the structural plane of good combination, average combination, and poor combination, the theoretical calculation results of the *Hrc* minimum values were 51.69 m, 30.79 m, and 14.05 m in sequence.

### The control effect of the rock structural plane strength

In order to explore the variation law of the self-stabilizing control effect of the rock structural plane strength on the FPVRW in layered rock stratum, the strength reduction coefficient (*k*) of rock structural planes was introduced, and synchronously reduced the cohesion force (*c*) and internal friction angle (*φ*) of the rock structural plane according to Eq. (9).

9 $$ c{\prime} = \frac{c}{{\text{k}}}\,\varphi{\prime} \, = \,\arctan \left( {\frac{\tan \varphi }{k}} \right) $$

**Figure 11:**
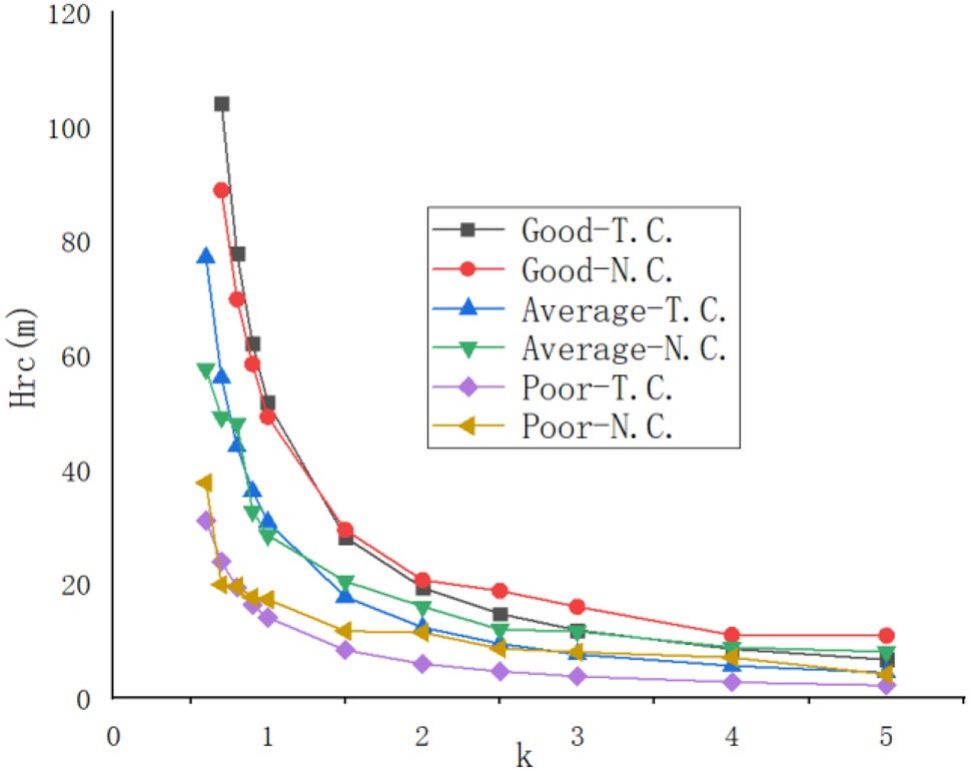
The change distribution characteristics of the *Hcr* varied with *k*.

The strength reduction coefficient (*k*) of the rock structural plane was taken as 0.6, 0.7, 0.8, 0.9, 1.0, 1.5, 2.0, 2.5, 3.0, 4.0, 5.0 in order, and all of the inclination angle α of the rock structural plane was determined taken as 60º. The distribution characteristics of the *Hcr* varied with the gradually increasing of *k* were shown in Fig. 11.

The conclusions could be drawn from Fig. 11 that the overall variation pattern of "continuously decreasing and rapidly decreasing first, then slowly decreasing and tending to stabilize" for the *Hcr* varied with the gradually increasing of the strength reduction coefficient *(k*) was presented. Further analysis reveals that for the deep foundation pit in layered rock stratum with the structural plane of good combination, average combination, and poor combination, when *Hcr* was 10 m, the corresponding *k* values were 3.7, 2.5, and 1.3 respectively.

## Discussion

There were many advantages in the study on the self-stabilizing control effect of the structural plane characteristics on the deep foundation pit vertical-rock-wall in layered rock stratum, such as it was beneficial for understanding and mastering the overall variation law of the self-stability characteristics of the FPVRW, determining the reasonable support structure of deep foundation pits, and predicting the safety risks of deep foundation pit construction et.al. Accurately determining the instability mode was the prerequisite and the foundation for reasonable calculation and analysis of the self-stability control effect of deep foundation pits. Jin, etc.^37,38^ studied the sliding failure law of bedding slopes in rock foundation pits with developed structural planes, and constructed corresponding stability and safety factor calculation formulas. Based on this, sensitivity analysis on the stability of rock foundation pit slopes was conducted on the influence of factors such as the spacing and inclination angle of parallel structural planes, the shear strength of rock mass structural planes, etc. However, the inclination angle of the rock mass structural plane analyzed above is limited to the range of 60–80°. It is not possible to reveal the complete variation law of the stability of the foundation pit with the inclination angle of the structural plane.This paper comprehensively discusses the stability of foundation pits with structural plane inclination angles in the range of 35–85°, and the complete curve of the variation law of the stability of the foundation pit with the structural plane inclination angle has been obtained. At present, it is difficult to comprehensively and accurately grasp the development status of the rock structural plane along the direction of the foundation pit, and it is extremely difficult to reasonably determine the strength parameters of the structural plane. In addition, the strength of the structural plane of rock deep foundation pits also exhibits strong time–space effects. Existed research results indicated that the influence of groundwater could reduce the shear strength to 30–70%, or even to 0 for rock masses with argillaceous cementitious structural planes. Therefore, the strength reduction problem of rock structural planes should be fully considered, and the self-stability control effect of the deep foundation pit should be accurately analyzed to ensure engineering safety during the excavation process of the foundation pit. At the same time, the safety reserve coefficient of the self-stability control effect of the FPVRW needs to be maintained.

In fact, the self-stabilizing control effect of the structural plane characteristics on the deep foundation pit vertical-rock-wall in layered rock stratum is a complex system engineering. It is not only closely related to the inclination angle and shear strength of the rock mass structural plane in which it is located, but also closely related to factors such as the direction, dip, connectivity, roughness, and filling characteristics of the rock mass structural plane. As well as it is also closely related to factors such as the excavation depth, shape, and surrounding environment of the foundation pit. In addition, it is closely related to factors such as the seismic characteristics of the area where the foundation pit is located and the blasting vibration during foundation pit construction. In the future, the research on the self-stabilization control effect of foundation pits can be conducted by combining dynamic characteristics such as earthquakes and blasting vibrations, as well as environmental characteristics around the foundation pit. At the same time, the further research can be conducted on the impact of rainfall, weathering, changes in groundwater level, and other factors on the strength of rock mass structural planes, as well as their impact on the self-stability control effect of foundation pits. Further enrich the research content, set the research background closer to engineering practice, so that the research results can better guide engineering practice.

## Conclusions

In this paper, multiple research methods such as model test, numerical calculation, and theoretical analysis were comprehensively utilized to study the self-stability control effect of the structural plane characteristics on the deep foundation pit vertical-rock-wall in layered rock strata. The main conclusions could be summarized as follows:

1. The instability mechanism of the deep foundation pit vertical-rock-walls in layered rock stratum was revealed by model tests. The entire process evolution law of the deep foundation pit vertical-rock-walls self-stable characteristics was obtained. The calculation equation of the deep foundation pit vertical-rock-wall self-stability critical height in layered rock stratum was constructed.
2. The overall variation trend of "Sharp decrease ~ Slow decrease ~ Slow increase ~ Sharp increase" in a symmetrical distribution for the *Hcr* varied with the gradually increasing of structural plane inclination angle was obtained. Meanwhile, the variation trend of "continuously decreasing and rapidly decreasing first, then slowly decreasing and tending to stabilize" with the gradually increasing of the structural plane strength reduction coefficient.
3. The viewpoint that the key to control the self-stability of the deep foundation pit vertical-rock-walls in layered rock stratum lied in fully grasping and utilizing the basic characteristics of rock structural planes such as their occurrence and development degree was proposed.

## Data Availability

The data used to support the findings of this study are available from the corresponding author upon request.
